# Supplementary material for: Antimicrobial Activity of Gelatin Nanofibers Enriched by Essential Oils against Cutibacterium acnes and Staphylococcus epidermidis
Source: Nanomaterials (Basel). 2023 Feb 24;13(5):844. doi: 10.3390/nano13050844 (PMC10005654; doi:10.3390/nano13050844)
Supplement: Supplementary file 1 [file nanomaterials-13-00844-s001.zip › nanomaterials-2223114-supplementary.pdf]

Table S1. Qualitative analysis obtained from GC/MS

RT	RI exp	RI lit	Compound	Lavender F Bulgaria	Lavender S Provence	Mint F Spain	Mint S India
5.67	1020	1026	α-Pinene	D	D	D	D
5.81	1027	1027	α-Thujene	D	D		
6.66	1068	1069	Camphene	D	D	D	D
7.71	1115	1111	β-Pinene	D	D	D	D
8.07	1129	1124	Sabinene	D	D	D	D
8.79	1157	1147	3-Carene	D	D	D	
9.24	1174	1161	Myrcene	D	D	D	D
9.6	1188	1157	4-Carene			D	
9.63	1190	1180	α-Terpinene				D
10.18	1210	1200	D-Limonene[s]	D	D	D	D
10.42	1219	1212	Eucalyptol	D	D	D	D
11.2	1249	1235	(Z)-β-Ocimene	D	D	D	D
11.45	1258	1246	γ-Terpinene			D	D
11.69	1267	1250	(E)-β-Ocimene	D	D	D	
11.96	1277	n.r.	Isolimonene			D	D
12.15	1284	1277	m-Cymene			D	D
12.2	1286	1298	o-Cymene	D	D		D
12.47	1296	1283	Terpinolene	D	D	D	D
13.91	1350	1355	6-Methylhept-5-en-2-one				D
14.23	1362	1354	1-Hexanol	D	D	D	
14.41	1369	1367	3-Nonanone			D	D
14.9	1387	n.r.	3.4-Dimethyl-2.4.6-octatriene	D			
15	1391	1379	1-Octen-3-yl acetate	D	D		
15.12	1396	1382	(Z)-3-Hexen-1-ol			D	D
15.29	1402	1393	3-Octanol	D	D	D	D
15.92	1427	1414	Hexyl butanoate	D	D		
16.2	1437	1429	(Z)-Thujone			D	D
16.67	1456	1437	p-Cymenene			D	D
16.82	1462	1450	1-Octen-3-ol	D	D		
17	1469	1449	Acetic acid	D	D		
17.35	1482	1473	Menthone			D	D
18.12	1512	1487	Menthofurane				D
18.33	1521	1528	(E)-p-Menthan-3-one			D	D
18.68	1535	1532	(+)-2-Bornanone	D	D		
18.77	1539	1526	β-Bourbonene			D	D
18.96	1547	1554	Menthyl acetate				D
19.2	1556	n.r.	p-Menth-4-en-3-one			D	D
19.32	1561	1547	Linalool[s]	D	D	D	
19.59	1572	1556	1-Octanol			D	
19.91	1585	1680	Linalyl butyrate	D	D		
20.14	1595	1597	Isopulegone			D	

20.23	1598	1576	β-Ylangene			D	
20.43	1607	1593	Bergamotene	D	D		
20.45	1608	1596	(E)-Isopulegone			D	
20.52	1611	1604	Isocaryophyllene		D		
20.61	1614	1576	α-Santalene	D	D		
20.66	1617	1636	Menthol			D	D
20.73	1620	1580	Bornyl acetate	D	D		
21.03	1632	1640	Terpinen-4-ol acetate	D	D		
21.13	1636	1606	Levandulyl acetate	D	D		
21.24	1641	1627	β-Caryophyllene	D	D	D	
21.35	1646	n.r.	hexyl (E)-2-Methylbut-2-enoate		D		
21.41	1648	1653	(Z)-β-Farnesene	D	D	D	D
21.82	1666	1587	Cedrene	D	D		
22.16	1680	1661	Pulegone	D	D	D	
22.22	1683	1676	ε-Muurolene			D	
22.3	1686	1665	(E)-β-Farnesene	D	D		
22.38	1689	1621	p-Menthan-1-ol			D	D
22.52	1695	1677	Levandulol	D	D	D	
22.61	1699	1679	Cryptone	D	D		
22.65	1701	1674	β-Humulene			D	D
22.71	1704	1663	(Z)-Verbenol	D	D	D	D
22.9	1712	1726	α-Muurolene			D	D
23.07	1720	1697	α-Terpineol	D	D	D	D
23.2	1725	1701	Borneol	D	D	D	D
23.42	1735	1541	β-Cubebene	D	D		
23.62	1744	1723	Neryl acetate	D	D		
23.82	1753	1733	α-Citral[s]	D	D		
23.95	1759	1738	Carvone	D	D	D	D
24.15	1768	1720	Dihydrocarveol			D	
24.3	1774	1781	1-Decanol			D	D
24.32	1775	1752	Geranyl acetate	D	D		
24.35	1776	n.r.	δ-Cadinene				D
24.41	1779	1765	Citronellol[s]			D	
24.45	1781	1765	γ-Cadinene	D	D		
24.87	1800	1794	p-Methylacetophenone	D	D	D	
25.02	1807	1814	Cuminaldehyde	D	D		
25.11	1811	1796	Myrtenol			D	
25.36	1823	1739	Linalool oxide (pyranoid)	D	D		
25.52	1830	1830	Buccocamphor			D	
25.68	1837	1830	2.6-Dimethyl-3.5.7-oktatrien-2-ol	D			
25.79	1843	n.r.	E-pinokarveol	D			
26	1852	1845	(E)-Carveol	D	D	D	D
26.19	1861	1845	Geraniol[s]	D	D		
26.33	1868	1852	p-Cymen-8-ol	D	D	D	D
26.76	1888	1889	(Z)-Myrtanol			D	D

26.83	1891	1870	Benzyl butanoate	D			
26.98	1898	1897	Benzyl alcohol[s]	D	D	D	
27	1899	1796	Myrtenol				D
27.14	1906	1896	1(7),8-p-Menthadien-2-ol			D	
27.72	1934	1905	2-Phenylethyl alcohol	D		D	D
28.18	1957	1963	3.7-Dimethyl-1.5-oktadien-3.7-diol	D	D		
28.32	1963	1961	(Z)-Jasmone			D	D
29.18	2003	1987	Caryophyllene oxide	D	D		D
29.53	2015	n.r.	(Z)-α-santalol	D			
30.1	2037	2009	p-Anisaldehyde			D	D
30.11	2038	2041	(E)-Nerolidol	D			
30.4	2044	2033	(E)-Cinnamaldehyde			D	
30.46	2046		Caryophyllenyl alcohol				D
306	2051	2080	Cubenol	D			
31.06	2066	2046	Viridiflorol				D
31.46	2079	2098	p-Cymen-7-ol	D	D		
31.83	2091	2128	2.6-Dimethyl-1.7-oktadien-3.6-diol	D	D	D	
32.71	2126	2170	τ-Cadinol	D	D		
32.76	2128	2166	Eugenol[s]			D	D
33.03	2139	2176	Thymol			D	D
33.5	21,58	2143	β-Bisabolole		D		
33.74	2168	n.r.	m-Cumenol	D			
35.43	2238	n.r.	Mintlactone			D	D
38.04	2347	n.r.	Coumarin[s]	D	D		
Total number of detected components				66	60	62	50

[*s]- standard, D – detected

Table S2. Agar diffusion tests of gelatin nanofibers with 20% addition of EOs

C. acnes	
Control agar plate	Lavender S Provence
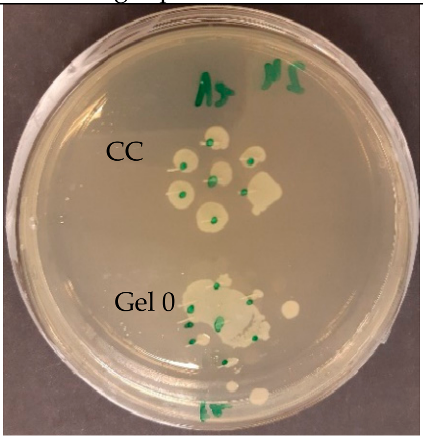	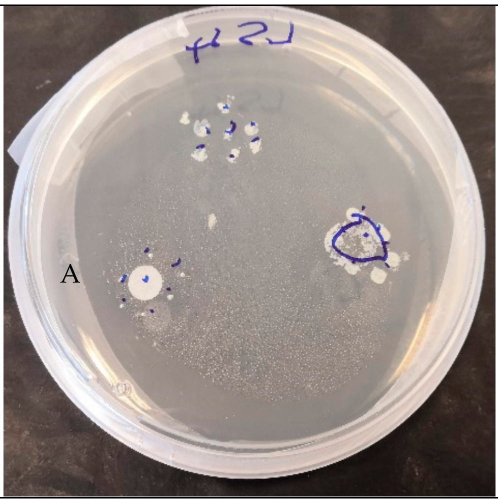
Mint F Spain	Mint S India
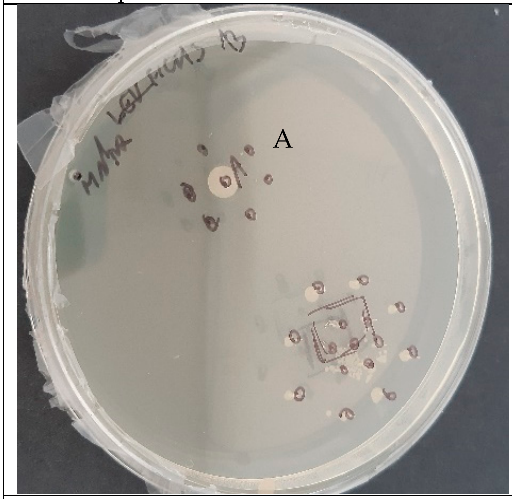	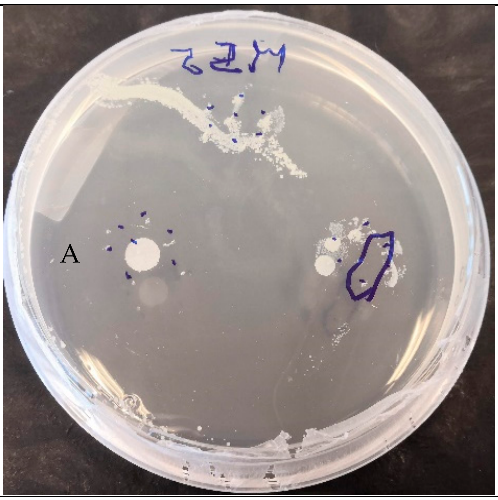
S. epidermidis	
Control agar plate - culture control spread on whole surface of the agar plate	Lavender S Provence
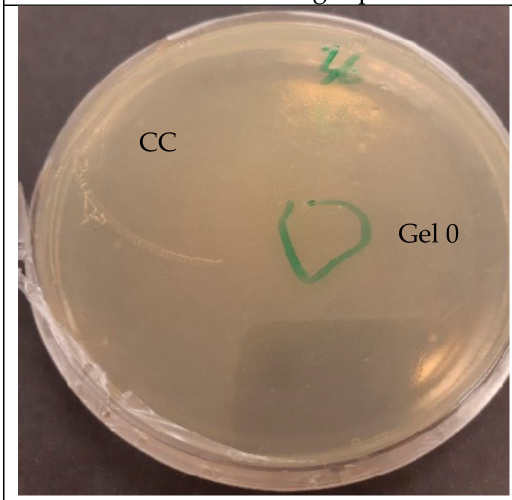	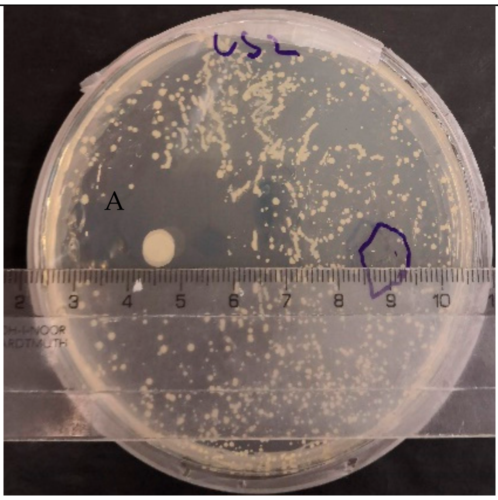
Mint F Spain	Mint S India

<table>
<tr><td>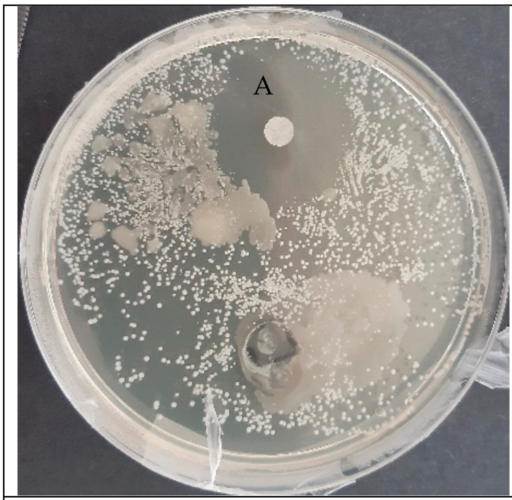
</td><td>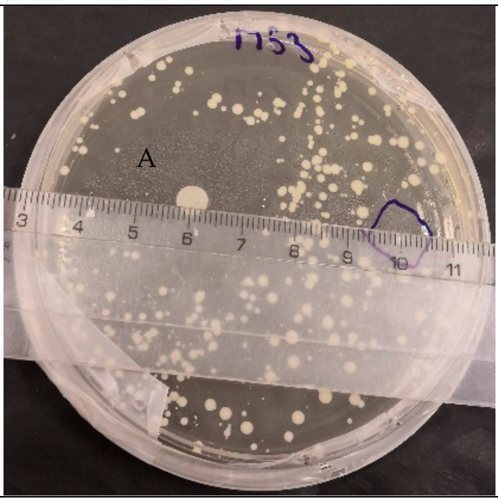
</td></tr>
<tr><td colspan="2">,</td></tr>
<tr><td></td><td></td></tr>
</table>

A – ampicillin 25 ug/mL, the marked area represents where nanofibers were placed, CC -culture control, Gel 0 – gelatin nanofibers with no added EOs
